# Transplanted Erythropoietin-Expressing Mesenchymal Stem Cells Promote Pro-survival Gene Expression and Protect Photoreceptors From Sodium Iodate-Induced Cytotoxicity in a Retinal Degeneration Model

**DOI:** 10.3389/fcell.2021.652017

**Published:** 2021-04-27

**Authors:** Avin Ee-Hwan Koh, Hiba Amer Alsaeedi, Munirah Binti Abd Rashid, Chenshen Lam, Mohd Hairul Nizam Harun, Min Hwei Ng, Hazlita Mohd Isa, Kong Yong Then, Mae-Lynn Catherine Bastion, Aisha Farhana, Mohammad Khursheed Alam, Suresh Kumar Subbiah, Pooi Ling Mok

**Affiliations:** ^1^Department of Clinical Laboratory Sciences, College of Applied Medical Sciences, Jouf University, Sakaka, Saudi Arabia; ^2^Department of Biomedical Sciences, Faculty of Medicine and Health Sciences, Universiti Putra Malaysia, Selangor, Malaysia; ^3^Department of Ophthalmology, Faculty of Medicine, Universiti Kebangsaan Malaysia Medical Centre, Kuala Lumpur, Malaysia; ^4^Tissue Engineering Centre, Universiti Kebangsaan Malaysia Medical Center, Kuala Lumpur, Malaysia; ^5^Department of Orthodontics, College of Dentistry, Jouf University, Sakaka, Saudi Arabia; ^6^Department of Medical Microbiology, Universiti Putra Malaysia, Serdang, Malaysia; ^7^Genetics and Regenerative Medicine Research Group, Universiti Putra Malaysia, Serdang, Malaysia; ^8^Centre for Materials Engineering and Regenerative Medicine, Bharath Institute of Higher Education and Research, Chennai, India

**Keywords:** mesenchymal stem cells, erythropoietin, sodium iodate, transcriptome, photoreceptors, pro-survival genes

## Abstract

Mesenchymal stem cells (MSC) are highly regarded as a potential treatment for retinal degenerative disorders like retinitis pigmentosa and age-related macular degeneration. However, donor cell heterogeneity and inconsistent protocols for transplantation have led to varied outcomes in clinical trials. We previously showed that genetically-modifying MSCs to express erythropoietin (MSC^EPO^) improved its regenerative capabilities *in vitro*. Hence, in this study, we sought to prove its potential *in vivo* by transplanting MSCs^EPO^ in a rat retinal degeneration model and analyzing its retinal transcriptome using RNA-Seq. Firstly, MSCs^EPO^ were cultured and expanded before being intravitreally transplanted into the sodium iodate-induced model. After the procedure, electroretinography (ERG) was performed bi-weekly for 30 days. Histological analyses were performed after the ERG assessment. The retina was then harvested for RNA extraction. After mRNA-enrichment and library preparation, paired-end RNA-Seq was performed. Salmon and DESeq2 were used to process the output files. The generated dataset was then analyzed using over-representation (ORA), functional enrichment (GSEA), and pathway topology analysis tools (SPIA) to identify enrichment of key pathways in the experimental groups. The results showed that the MSC^EPO^-treated group had detectable ERG waves (*P* <0.05), which were indicative of successful phototransduction. The stem cells were also successfully detected by immunohistochemistry 30 days after intravitreal transplantation. An initial over-representation analysis revealed a snapshot of immune-related pathways in all the groups but was mainly overexpressed in the MSC group. A subsequent GSEA and SPIA analysis later revealed enrichment in a large number of biological processes including phototransduction, regeneration, and cell death (*P*_adj_ <0.05). Based on these pathways, a set of pro-survival gene expressions were extracted and tabulated. This study provided an in-depth transcriptomic analysis on the MSC^EPO^-treated retinal degeneration model as well as a profile of pro-survival genes that can be used as candidates for further genetic enhancement studies on stem cells.

## Introduction

Regenerative medicine is a fast-paced field that has contributed heavily to stem cell research. A variety of stem cells have been utilized in clinical trials to replace wounded tissue and treat many degenerative diseases. Mesenchymal stem cells (MSCs) are highly potent cells that are not heavily restricted by ethical guidelines, such as those involving embryonic (Mintrom, 2013) and fetal stem cells (Ishii, 2014). But like other cell-based therapies, MSC treatment is presented with its own set of limitations. Cell donor heterogeneity arising from epigenetic modifications and health conditions can result in varied clinical benefits that reduce the overall effectiveness of the treatment (McLeod and Mauck, 2017; Kang et al., 2018; Lee, 2018). As a result, younger donors tend to have more robust cells compared to older donors. Although allogeneic transplants are an option for older patients, the risks of graft rejection also cannot be ignored (Trounson and McDonald, 2015). Apart from these, inconsistent transplantation procedures and poor cell engraftment is also a common issue (Kean et al., 2013; Baldari et al., 2017; Ezquer et al., 2017). Hence, researchers have developed a workaround solution by pre-treating MSCs with growth factors/cytokines to stimulate cellular proliferation, growth, and enhance regenerative potential (Kavanagh et al., 2015; Wang et al., 2019). Erythropoietin (EPO) is one such factor that is known to exert anti-apoptotic (Lappin, 2003; Wang et al., 2015) and proliferative (Gawad et al., 2009; Günter et al., 2013) properties through its binding with non-erythroid EPO receptors (EPOR). Additionally, EPO can interact with MSCs to promote the secretion of microvesicles for stronger paracrine wound healing (Wang et al., 2015). It also confers a protective effect on these cells in the harsh microenvironment of wounds, thereby improving graft survivability (Ercan et al., 2014; Ding et al., 2018). As such, we sought to genetically enhance MSCs by combining them with EPO as a single treatment via genetic modification. This could result in an improved therapeutic outcome. To date, genetically modifying MSCs to express EPO is not widely explored.

In this study, we aimed to investigate the therapeutic potential of MSCs^EPO^ in a rat retinal degeneration model that was established using NaIO_3_ (Koh et al., 2019). Firstly, MSCs^EPO^ were cultured and expanded until the cells were sufficient enough for intravitreal transplantation into the sodium iodate-induced model. Once the procedure was done, electroretinography (ERG) was performed bi-weekly for 30 days. Histological analyses were then performed at the end of the time-point analysis. After that, the retina samples were harvested for RNA-Seq. Salmon and DESeq2 were used to process the data for downstream applications. The generated dataset was analyzed using over-representation analysis (ORA), functional enrichment analysis (GSEA), and pathway topology analysis (SPIA) tools to identify key enriched pathways, such as phototransduction and cell death. Based on these pathways, a set of pro-survival gene expressions were able to be extracted and tabulated. In this study, we provided an in-depth transcriptomic analysis on the MSC^EPO^-treated retinal degeneration model and also revealed a unique profile of genes that likely contributed to the survival of retinas exposed to sodium iodate.

## Materials and Methods

### Animal Handling and Ethics

The study was conducted in full compliance with the rules and regulations set by the Universiti Kebangsaan Malaysia Animal Ethics Committee (UKMMAEC) (UKM FPR.4/244/FF-2014-376). Adult Sprague-Dawley rats (*N* 28), aged at post-natal weeks 8–9, were used in this study. The rats were reared in Individually Ventilated Cages (IVC) under a strict 12 h light and dark cycle throughout the study except during overnight dark adaptation prior to an electroretinography test.

### Culture and Expansion of MSCs and MSCs^EPO^

The mesenchymal stem cells used in this study were harvested from human Wharton’s jelly. In brief, the Wharton’s jelly was rinsed in fresh phosphate-buffered saline (PBS) and transferred into T25 tissue culture flasks containing DMEM/F12 medium (Invitrogen, CA, United States) supplemented with 10% fetal bovine serum (Invitrogen, CA, United States) and 1% penicillin-streptomycin (Invitrogen, CA, United States). The culture was incubated in a humidified cell culture incubator at 37°C, 5% CO_2_. After reaching 80% confluency, the flasks were rinsed with PBS and the adherent cells were harvested with 0.25% trypsin-ethylene diamine tetra acetic acid (EDTA) (Invitrogen, CA, United States). The process was repeated to remove any remaining floating cells. In brief, 1 × 10^6^ MSCs were transferred from an ampoule into a 15 ml tube with 10 mL of complete culture medium. The cells were centrifuged at 1,000 × *g* for 10 mins and resuspended in complete culture medium. The cells were then plated at 3,000 cells/cm^2^ in plastic tissue culture flasks and incubated in a humidified cell culture incubator at 37°C, 5% CO_2_. Characterization of MSCs has been published in our previous studies (Ding et al., 2018, 2019). The characterization included immunophenotyping and differentiation into the three mesodermal lineages. In brief, immunophenotyping was performed to identify the positive expression of the key MSC markers CD90, CD73, CD105, CD29, CD44, and HLA-ABC. Several non-MSC markers were also tested to ensure MSC purity, and this included CD34, CD14, CD45, CD80, and CD86, which were not detected. Cytochemical staining was performed to confirm the differentiation into adipogenic, chondrogenic, and osteogenic lineages. The previously characterized MSCs were used in the present study. The genetic modification of MSCs^EPO^ was performed in our previous study at Ding et al. (2019). In short, lentiviral particles containing the pReceiver-Lv183 plasmid (GeneCopoeia, MD, United States) and human *EPO* gene (NCBI accession number: NM_000799.2) was used to transduce the MSCs (P3 – 6). The plasmid contained a GFP marker immediately downstream of the EPO construct. The efficiency was up to 11.9%, as measured with flow cytometry. Following that, sorting was performed using CD44-specific antibodies (BD Biosciences, CA, United States) and GFP as expression markers with the FACSAria III system. Up to 90% MSCs^EPO^ were sorted and re-characterized (Ding et al., 2018, 2019). The sorted cells were plated at 6,000 cells/cm^2^ in plastic tissue culture flasks with complete culture medium. Measurement of EPO secretion was also performed in our previous study (Ding et al., 2018). In brief, MSCs^EPO^ were grown in complete culture medium for 72 h. The conditioned medium was then collected and filtered through a 0.22 μM syringe filter. The ELISA was then performed according to the manufacturer’s protocol, whereby 450 nm was used to obtain the optical density values for estimating EPO concentration based on the standard curve. The concentration was approximately 110 mIU/ml from an initial seeding density of 3,000 cells/cm^2^.

### Intravitreal Transplantation

Intravitreal injection of stem cells was performed as described by Ji et al. (2018) 4 days before the induction of retinal degeneration. MSCs and MSC^EPO^ were trypsinized, centrifuged, and resuspended in Hank’s balanced saline solution (HBSS) (Invitrogen, CA, United States) to achieve a final injection concentration of about 1 × 10^4^ cells/μl. Before loading into the syringe, 10 μl of the cells were first subjected to gentle mechanical dissociation. Then, 2 μl of the cell suspension was loaded into a sterilized Hamilton syringe once the operation was ready to minimize cell clumping (Leow et al., 2015). During the transplantation, the rats were first anesthetized with an intramuscular administration of ketamine (80 mg/ml) and xylazine (8 mg/ml) at a dose of 0.001 ml/g. The pupils were then dilated with Mydriacyl (1% tropicamide) (Alcon, TX, United States). Alkaine (0.5% proparacaine hydrochloride) (Alcon, TX, United States) anesthetic eyedrops were then given. During the injection, a tract was formed by carefully puncturing the right cornea with a sharp insulin needle. After that, 2 μL of cells were slowly deposited with the blunt Hamilton syringe. To avoid excessive leakage of cells, the syringe was carefully withdrawn 30 s later. Maxitrol antibiotic eyedrops (1 mg dexamethasone, 6000 IU polymyxin B sulfate, and 3500 IU neomycin sulfate) (Alcon, TX, United States) were then applied to the eyes. There was a total of 4 groups in the study, i.e., healthy control (N = 8), NaIO_3_-administered vehicle/sham control (N = 8), MSC (N = 6), and MSC^EPO^-transplanted (N = 6) groups. In the vehicle control group, HBSS was injected without cells. After the operation, the rats were observed daily for any signs of surgical complications. Dexamethasone immunosuppressant (Novartis, Basel, Novartis) was injected intraperitoneally at a 2 mg/kg dose for 3 days before transplantation. After transplantation, it was replaced with cyclosporin A-infused (Novartis, Basel, Switzerland) drinking water (100 μg/mL). This was given *ad hoc* until day 30 of the study.

### Sodium Iodate Degeneration Model

Retinal degeneration in post-transplanted (4-day) Sprague-Dawley rats (herein designated as day 0 in the present study) were induced using a systemic administration of sodium iodate (NaIO_3_) as per our previous study (Koh et al., 2019). The compound was prepared fresh by diluting NaIO_3_ (Alfa Aesar, MA, United States) in sterile Hank’s balanced salt solution (10% w/v) and stored at RT before administration. Prior to the administration, the rats were completely anesthetized and rested on warm heating pads. The compound was then injected systemically via the caudal vein at a dose of 40 mg/kg.

### Electroretinography Test

Electroretinography was conducted bi-weekly post-operation until the end of the study on day 30. Before it was carried out, the rats were dark-adapted overnight. Electroretinography was conducted in a dark room throughout the test. Firstly, the rats were completely anesthetized. The pupils were dilated with Mydriacyl (1% tropicamide) (Alcon, TX, United States), and Alkaine (0.5% proparacaine hydrochloride) (Alcon, TX, United States) anesthetic eyedrops were then given. Silver chloride loop electrodes were placed on each eye, while the reference and ground electrodes were attached to the ear and tail, respectively. After carefully wheeling the rat onto the platform of the RETI-port Roland Consult ERG instrument, the test was initiated. Five light intensities were used for the full-field scotopic measurement, i.e., the 0.003, 0.03, 0.03 (9 hz flicker), 0.3, and 3.0 cd.s/m^2^ flash intensities. An average of 10–20 flashes per intensity was averaged and processed using the RETI-port system.

### Enucleation, Histology, and Immunohistochemistry

At the end of the study, the rats were euthanized with an overdose of sodium pentobarbitol (150 mg/kg) (Zoetis, NJ, United States). The eyeballs were immediately enucleated by excising the surrounding connective tissue and then snap-frozen in liquid nitrogen. The eyes were then dissected using a cryostat (Sakura, CA, United States) into sections of 4 microns. General hematoxylin & eosin staining was performed on several sections while immunohistochemistry was performed on several others, as described by Koh et al. (2019). For quantitative histological analysis of transplanted retinas, visual fields along the section were taken to measure the thickness of the entire retina. This included the peripheral and central retina. For immunohistochemistry, the primary antibody used was anti-STEM121 (1:400) (Takara Bio, Japan), while DAPI acted as the counterstain. The antibodies (in 1% BSA solution) were added to the sections and incubated overnight at 4°C. During the next day, the slides were washed thrice with PBS solution. Then, secondary antibodies were added and incubated for 1 h (AF594, 1:400) (Abcam, United Kingdom). After three more washing steps with PBS, the coverslips were mounted with a DAPI containing mounting medium and viewed under a fluorescence microscope.

### RNA Extraction and NGS Library Preparation

Once the eyes were harvested, the retinas were isolated and immediately incubated in RNAlater (Qiagen, Germany) at 4°C overnight. After centrifuging and removing excess RNAlater the next day, the tissues were stored at −80°C or processed immediately. RNA extraction was carried out using the RNeasy Plus Universal Kit (Qiagen, Germany) according to the manufacturer’s protocol. In brief, the tissue was lysed in QIAzol lysis reagent and mixed with a gDNA Eliminator solution. After centrifuging the mixture at maximum speed for 15 min at 4°C, the resulting upper aqueous layer was transferred to a fresh tube containing 70% ethanol at an equal volume. The samples were then spun through an RNeasy Mini Spin column, washed several times, and finally eluted into a fresh RNase-free tube with 50 μL of RNase-free elution buffer. Quality control was then performed with the Qubit fluorometer (Thermo Fisher Scientific, MA, United States) and Bioanalyzer 2100 system (Agilent Technologies, CA, United States). All samples (N = 3 per group) had RIN ≥ 8.5. NGS library construction was performed using the QIAseq Stranded mRNA Select kit (Qiagen, Germany) according to the manufacturer’s protocol. In brief, 15 samples were mRNA-enriched using 1 μg of total RNA. The mRNA samples were fragmented and converted to cDNA. First and second-strand syntheses were then performed before the samples were barcoded with Illumina-compatible adapters. Once the libraries were constructed, another quality control check was performed with the Qubit fluorometer and Bioanalyzer 2100 system. The libraries were pooled and from that, 1.6 pM was transferred for RNA-seq using the NextSeq 500 system (Illumina, CA, United States). Paired-end sequencing was performed (2 × 75 bp) for a minimum of 25 million expected reads per sample.

### RNA-Seq Data Quality Control, Alignment, and Quantification

Upon completion of RNA-Seq, the de-multiplexed output files were concatenated to yield individual forward and reverse FASTQ files per library (91% sequence quality ≥ Q30, average no. of reads = 55 million). The FASTQ output files were then examined using FastQC (version 0.11.9) in interactive mode. No Illumina adapter contamination was present after trimming, and the data was of good quality. Selective alignment was then performed by mapping the reads to the *Rattus norvegicus* transcriptome and genome assembly from Ensembl (Rnor_6.0) using Salmon (version 1.1.0)(Srivastava et al., 2020). A k-mer of 31 was used for the construction of the reference index. After that, transcript abundance values were converted to the gene level using Tximport (version 3.12) to generate an input file for normalization using DESEq2 (Love et al., 2014). The data were transformed using the regularized-logarithm (rlog) function. Annotation was done based on the Ensembl Rnor_6.0 assembly.

### Exploratory Data and DEG Analysis of RNA-Seq Data

Exploratory data analysis was performed after normalization with DESeq2. The pcaExplorer tool was used to construct a principal component analysis PCA plot using the rlog-transformed dataset for data visualization. Hierarchical clustering was performed after pairwise correlation using Pearson’s correlation coefficients. Differential gene expression analysis was performed using DESeq2, where *P*_adj_ < 05 and fold change > ± 2.0 were set as the arbitrary cutoff threshold statistical significance. Volcano plots were constructed for data visualization in R. An over-representation analysis was performed with the online g:Profiler tool (Raudvere et al., 2019) using the list of DEGs generated from DESeq2 to obtain a snapshot of top clustered transcriptomic profiles. The REViGO tool was then used to summarize and present a representative subset of these GO biological processes (Supek et al., 2011).

### Functional Enrichment and Pathway Topology Analysis

Functional enrichment analysis was performed using the Gene Set Enrichment Analysis (GSEA) tool (version 4.1.0)(Subramanian et al., 2007). The expression dataset, phenotype labels, and gene set files were prepared with the rlog-transformed dataset and then loaded into GSEA in the graphical interface mode. The metric used in gene ranking was ‘Signal2Noise’ and the number of gene set permutations was set to 1000. Gene annotation was performed using the Ensembl platform in GSEA (Rat_ENSEMBL_Gene_ID_Human_Orthologs_MSig DB.v7.2. chip). *Rattus norvegicus* (Rnor_6.0 assembly) was used as the reference genome. The Gene Ontology biological processes gene set from GSEA MsigDB was used as the database for functional enrichment. Other than these settings, the remaining fields were left at default. Pathway topology analysis was performed with the Signaling Pathway Impact Analysis (SPIA) tool (version 3.12) (Ihnatova et al., 2018). KEGG pathways were used as the reference database. Results with *P*_adj_ < 0.05 were considered statistically significant.

### Statistical Analysis

Histological measurements were performed with one-way ANOVA and Dunnett’s multiple comparisons test post-hoc. Electroretinographic comparisons were calculated using two-way ANOVA, followed by Tukey’s multiple comparisons test post-hoc. Prism 6 (GraphPad, CA, United States) was used for graphing (mean SEM) and statistical analyses apart from RNA-Seq analyses, in which case DESeq2, GSEA, and SPIA were used. All analyses involving DEGs and enrichment were considered statistically significant when *P*_adj_ < 0.05.

## Results

### Identifying Intravitreally-Transplanted MSCs^EPO^ in the Retinal Degeneration Model

**FIGURE 1:**
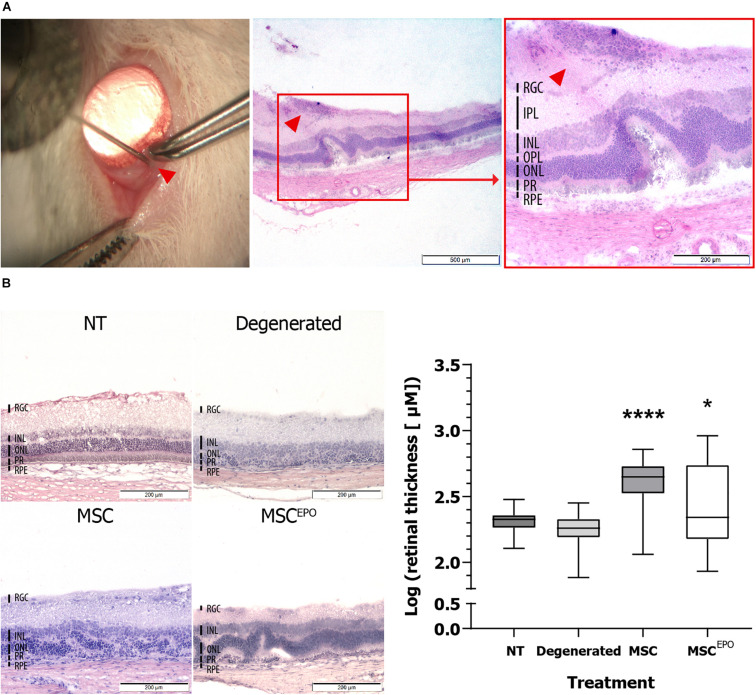
Ocular histology of normal, degenerated MSC, and MSC^EPO^-treated rats at day 31. **(A)** On day 31 post-operation, MSC^EPO^ transplanted sections were stained with H&E and a population of cells was found on the RGC layer. The sections were viewed under 40× and 100× total magnification. The scale bars denoted 500 and 200 μm, respectively. The indicator points to the site of injection. **(B)** By comparing the sections, thicker retinas were found in the stem cell-treated groups. The sections were viewed under 100× total magnification. The scale bars were denoted as 200 μm. The results were presented as mean ± SEM. *P* values were obtained using one-way ANOVA and Dunnett’s multiple comparisons test post-hoc (*P < 0.05, ****P < 0.001). NT, no-treatment control; Degenerated, NaIO_3_-only/vehicle control; RPE, retinal pigment epithelium; PR, rods and cons layer; ONL, outer nuclear layer; OPL, outer plexiform layer; INL, inner nuclear layer; IPL, inner plexiform layer; RGC, retinal ganglion cells.

Enucleation was performed on day 31 (after the study) for cryosectioning to identify intravitreally-transplanted stem cells in the model. This was done using H&E and immunohistochemical staining. On day 31, H&E staining of MSC^EPO^-transplanted sections revealed clusters of cells that have adhered to the retina. Upon closer inspection of the tissue, these cells were grafted on the retinal ganglion cell (RGC) layer (Figure 1A). The retinal thickness was quantified and compared between sections of healthy, sham (NaIO_3_-administered), MSC, and MSC^EPO^-transplanted groups. Statistically, the two latter groups of sections were significantly thicker than the controls (*P* <0.05) (Figure 1B).

**FIGURE 2:**
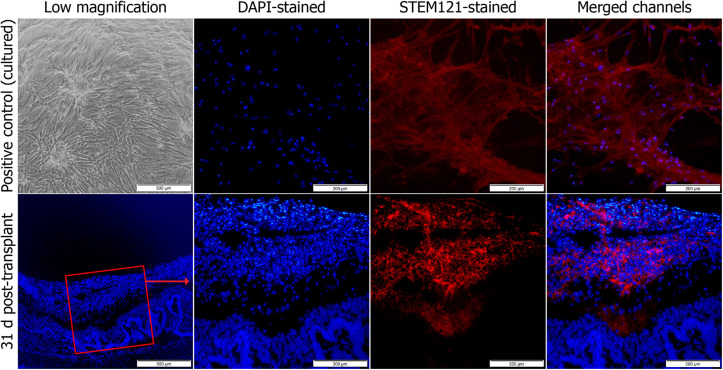
Immunohistochemical staining of transplanted MSCs^EPO^ in the retinal degeneration model. The stem cells were stained with human STEM121 antibodies (red signal), while DAPI (blue) was used as a nuclear counterstain. This was performed at the end of the study. After 30 days, the cells had engrafted onto the retina with no signs of penetration into the inner layers. Stained MSCs^EPO^ in culture was used as a positive control. The sections were viewed under 40× (low) and 100× total magnification. The scale bars were denoted as 500 and 200 μm, respectively.

To identify these adhered cells, immunohistochemistry was performed. Sections obtained on day 31 after the study were stained with STEM121 antibodies, which are specific to human cells (Figure 2). The cells were found grafted onto the retina.

### Stem Cell-Transplanted Models Had Stronger ERG Responses Compared With the Sham-Treated Group

**FIGURE 3:**
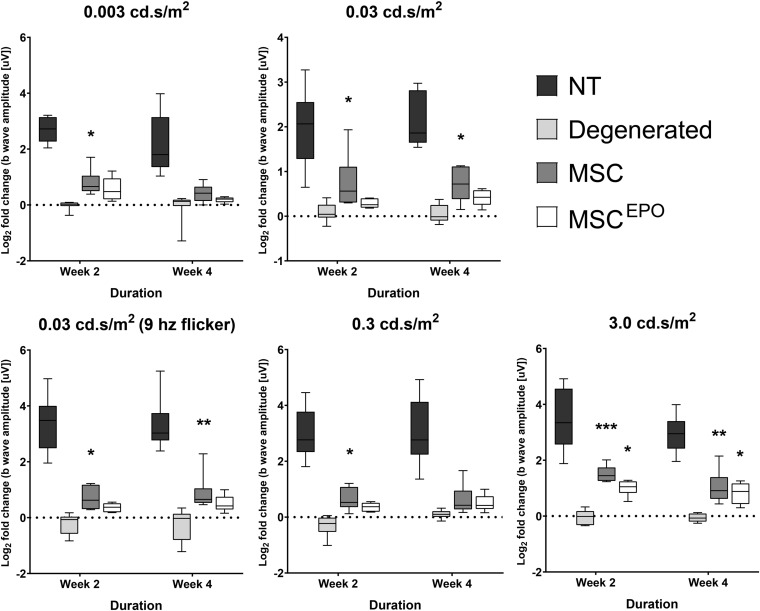
Electroretinographic readings of stem cell-transplanted retinal degeneration models in the study. An ERG test was performed on week 2 and week 4 to assess the effectiveness of MSC and MSC^EPO^ in protecting the retina from sodium iodate-induced damage. Five flash intensities were used (0.003, 0.03, 0.03–9 hz flicker, 0.3, and 3.0 cd.s/m2) for the recording. Rats transplanted with MSCs showed strong ERG responses in week 2 but began to weaken by week 4. Conversely, although the effectiveness of MSC^EPO^ was only detected with the brightest flash intensity, the responses were more consistent throughout the study. The results were presented as mean ± SEM. Two-way ANOVA, followed by Tukey’s multiple comparisons test post-hoc, was used to obtain the *P* values (*P < 0.05, **P < 0.01, ***P < 0.001). NT, no-treatment control; Degenerated, NaIO_3_-only/vehicle control; MSC, MSC-transplanted group; MSC^EPO^, MSC^EPO^-transplanted group.

An electroretinography test was conducted bi-weekly during the study until day 30. A total of 5 different flash intensities were used to invoke electrophysiological responses in the retina of the models (0.003, 0.03, 0.03–9 hz flicker, 0.3, and 3.0 cd.s/m^2^). Expectedly, healthy (NT) rats elicited strong ERG responses (Figure 3). These were recorded using the ERG b wave as it is the most clinically relevant indicator that represents the major component of the graph. Compared with the NT group, the NaIO_3_-only sham (vehicle control) group had much weaker b wave amplitudes. On the other hand, rats transplanted with MSCs showed stronger ERG responses when compared with the sham group. The b waves were more apparent in all the tested flash parameters. Strong responses were recorded in week 2, and the brightest flash intensity (3.0 cd.s/m^2^) produced the highest b waves. However, this began to slowly plummet by week 4. Conversely, although the MSC^EPO^ group also showed improved ERG responses, its effectiveness was only detected with the brightest flash intensity. Interestingly, the b wave responses in the MSC^EPO^-treated group were more consistent throughout the study when compared with the MSC-treated group, which showed declining ERG responses over time.

### Performing an Exploratory Data Analysis of RNA-Seq Count Data

**FIGURE 4:**
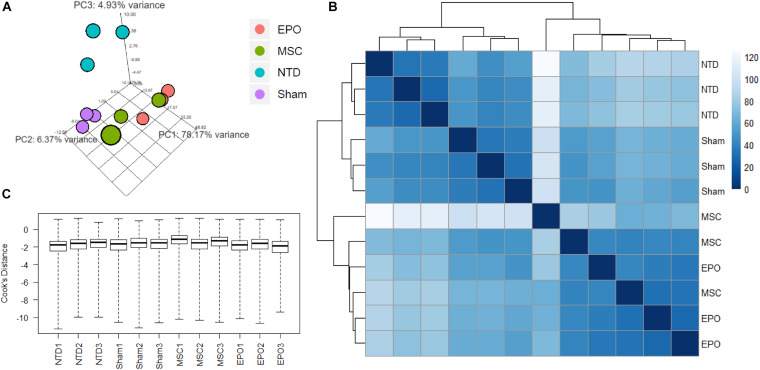
Exploratory data analysis of RNA-Seq count data was performed by measuring sample distances to evaluate for similarities. **(A)** A principal component analysis (PCA) plot was constructed to show variances of principal components among all the samples. The no-treatment and sham-treated samples showed high intra-group similarities and thus formed 2 distinct groups. **(B)** Hierarchical clustering was performed using DESeq2 based on Poisson distance, and the data was plotted as a sample-to-sample heatmap. The colored indicator represents sample distances (blue signifies a high correlation). Here, the clustering of samples and groups was more differentiated. The MSC and MSC^EPO^ groups showed a certain degree of similarity with each other. **(C)** A Cook’s distance boxplot was constructed to visualize the data after outlier detection and removal by DESeq2. NTD, no-treatment/healthy control; Sham, NaIO_3_-only/vehicle control; MSC, MSC-transplanted group; MSC^EPO^, MSC^EPO^-transplanted group.

Before analyzing the RNA-Seq data for gene expression studies, an exploratory data analysis was carried to assess data quality and explore sample correlations. Firstly, a principal component analysis (PCA) plot was constructed to identify sample grouping (Figure 4A). Expectedly, the no-treatment samples formed a clear cluster. Likewise, this was also shown for the sham samples, and both of these groups could be distinguished. In the case of the experimental groups, both MSC and MSC^EPO^ samples shared a higher correlation with each other when compared with the controls. However, although the MSC^EPO^ samples had a distinct cluster, the MSC samples showed a higher intra-group variance. A sample-to-sample distance matrix was then constructed based on Poisson distance (Figure 4B). Similar to the PCA plot, the no-treatment and sham groups showed clear grouping. Both the MSC and MSC^EPO^ samples were also shown to have a closer relationship with each other, which was expected since both cells shared the same source. One MSC sample, in particular, had a larger sample distance and was likely due to strong gene expressions which may be constituted as outliers. To tackle this, DESeq2 was used in the differential gene expression step to eliminate these factors. After outlier removal, the data was visualized in the form of a Cook’s distance boxplot for manual inspection of further outliers (Figure 4C). The samples were shown to have a comparable Cook’s distance distribution after processing.

### Differential Gene Expression Analysis Revealed Strong Expressions of Upregulated DEGs

**FIGURE 5:**
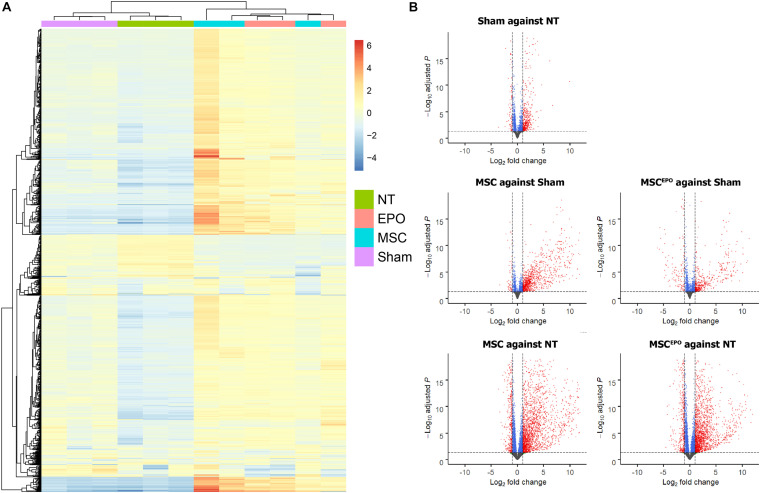
Differential gene expression analysis of normalized count data. **(A)** A heatmap was constructed to show hierarchical clustering of the top 1000 genes with the highest variance across all biological replicates. **(B)** After differential gene expression analysis was carried out, volcano plots were constructed to visualize the DEGs. Genes that have passed the threshold were represented as red dots (*P*_adj_ < 0.05, fold change >± 2.0). About 1000 DEGs were over and underexpressed in the sham group when compared with the healthy control. By using sham as the reference, 1559 overexpressed and 634 underexpressed DEGs were detected in the MSC group. The MSC^EPO^ group had 1009 overexpressed and 847 underexpressed DEGs. By using NT as the reference, both MSC and MSC^EPO^ groups were found to have more than 3000 DEGs that were over and underexpressed. NT, no-treatment/healthy control; Sham, NaIO_3_-only/vehicle control; MSC, MSC-transplanted group; MSC^EPO^, MSC^EPO^-transplanted group.

A differential gene expression analysis was performed using DESeq2 to investigate the effects of MSC and MSC^EPO^ transplantation on the degenerating retinas. Firstly, hierarchical clustering was done and a heatmap of the top 1000 genes with the highest absolute variance across all biological replicates was constructed (Figure 5A). After DEG analysis was performed, a total of 19638 genes were detected. To show a contrast between the groups as well as correlate the gene expression data with its respective false discovery rate threshold (*P*_adj_/FDR), volcano plots were constructed (*P*_adj_ < 0.05, fold change > ± 2.0) (Figure 5B). Red dots denote genes that have passed both filters. At first glance, NaIO_3_ administration led to a general upregulation of DEGS when compared with the healthy control. This uphill trend was further amplified in the MSC and MSC^EPO^-treated groups. By applying the threshold, about 1000 DEGs were over and underexpressed in the sham group when compared with the healthy control. Then, by using the sham group as the reference, 1559 overexpressed and 634 underexpressed DEGs were detected in the MSC-treated group. The MSC^EPO^ group had 1009 overexpressed and 847 underexpressed DEGs. The study was focused on the comparison of these aforementioned pairs of groups.

Next, an over-representation analysis was performed using the generated list of DEGs from DESeq2 and the g:Profiler tool. This provided a screenshot of the transcriptomic events that transpired after the stem cell transplant. After using REViGO to reduce GO term redundancy, the top GO terms on biological processes were then tabulated (Table 1). Based on the table, a majority of them were revealed to be immune-associated terms. This was similar to the ORA analysis performed for the other groups, and this made the identification of key pathways challenging. Thus, a separate strategy was employed, whereby the Gene Set Enrichment Analysis (GSEA) tool was used to reduce term redundancy and further scrutinize the expression data.

**TABLE 1:** Over-representation analysis on the list of DEGs was performed using g:Profiler and then summarized using REViGO. The top 5 representative GO biological processes were tabulated (*P* ≤ 0.05).

Source	GO term	GO ID	log_10_*P*
Sham treated group	Cell activation	GO:0001775	−16.1209
	Immune effector process	GO:0002252	−28.7471
	Immune system process	GO:0002376	−27.3344
	Inflammatory response	GO:0006954	−18.2464
	Biological adhesion	GO:0022610	−4.1749
MSC-treated group	Cell activation	GO:0001775	−128.6517
	Cell killing	GO:0001906	−19.2612
	Leukocyte mediated cytotoxicity	GO:0001909	−16.2716
	Immune system process	GO:0002376	−240.2358
	Positive regulation of immune system process	GO:0002684	−139.0975
MSC^EPO^-treated group	Cell activation	GO:0001775	−30.2857
	Immune system process	GO:0002376	−47.4776
	Positive regulation of immune system process	GO:0002684	−32.7328
	Biological adhesion	GO:0022610	−15.3316
	Developmental process	GO:0032502	−9.2976

### Functional Enrichment Analysis With GSEA Revealed Upregulated Healing and Regenerative Processes in MSC^EPO^ Treated Retinas

**FIGURE 6:**
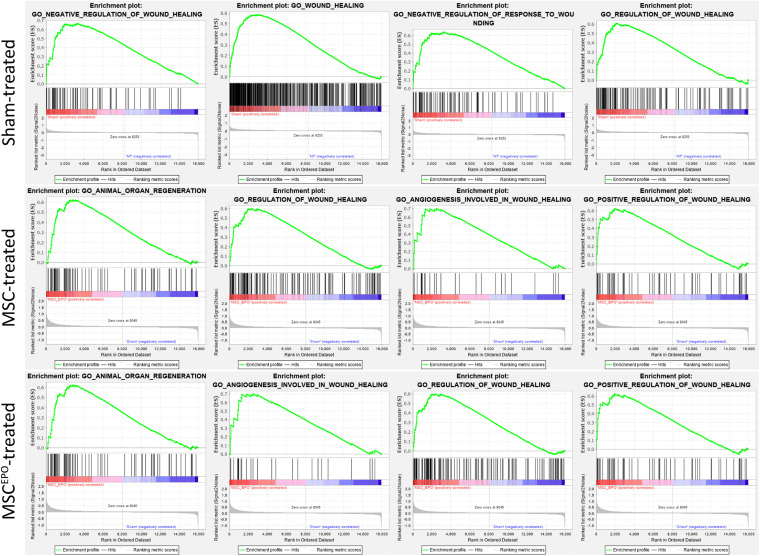
Functional enrichment analysis with GSEA revealed the impact of MSCs and MSCs^EPO^ on the healing capability of stem cell-treated retinas. Using GO biological processes as a reference database, enrichments in healing and regeneration were detected. The sham-treated group exhibited an enrichment involving mainly negative regulation of wound healing while regeneration and positively regulated healing gene sets were enriched in the MSC and MSC^EPO^-treated groups (*P*_adj_ < 0.05).

Functional enrichment analysis was performed using the Gene Set Enrichment Analysis tool to identify gene sets or pathways that have been enriched in the expression data. The scope was narrowed down to biological processes annotated by Gene Ontology in order to identify enrichment of profiles involved in healing and regeneration. Rats that were administered with NaIO_3_ exhibited an enrichment of gene sets that mainly involved negative regulation of wound healing (Figure 6). The normalized enrichment scores of the GSEA plots were tabulated in Table 2. In the MSC and MSC^EPO^-treated groups, there was an enrichment of regeneration and positively regulated wound healing gene sets. To determine whether these effects of transplanted MSCs and MSCs^EPO^ benefited the host, a pathway topology analysis was then carried out.

**TABLE 2:** After performing a GSEA analysis, the nominal enrichment scores of the enriched gene sets in healing and regeneration were tabulated along with the respective GO IDs (*P*_adj_ < 0.05). NES, nominal enrichment score.

Source	GO term	GO ID	NES
Sham treated group	Negative regulation of wound healing	GO:0061045	1.93
	Wound healing	GO:0042060	1.92
	Negative regulation of response to wounding	GO:1903035	1.90
	Regulation of Wound healing	GO:0061041	1.89
MSC-treated group	Animal organ regeneration	GO:0031100	1.60
	Regulation of wound healing	GO:0031100	1.59
	Angiogenesis involved in wound healing	GO:0031100	1.58
	Positive regulation of wound healing	GO:0090303	1.57
MSC^EPO^-treated group	Animal organ regeneration	GO:0031100	1.60
	Angiogenesis involved in wound healing	GO:0031100	1.60
	Regulation of wound healing	GO:0031100	1.60
	Positive regulation of wound healing	GO:0090303	1.55

### Pathway Topology Analysis Reveal the Activation of Pathways Involved in Phototransduction and Environmental Signaling as Well as Expressions of Pro-survival Genes

**FIGURE 7:**
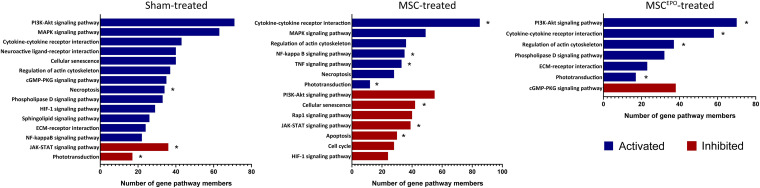
Pathway topology analysis was performed using SPIA with KEGG as the reference database. Phototransduction, which was one of the key pathways of this study, was found to be inhibited in the sham group. In both the MSC and MSC^EPO^ group, however, it was activated. Other notable pathways that were statistically significant in this analysis include necroptosis, apoptosis, JAK-STAT, NF-κB, TNF, and cytokine-cytokine receptor interactions. Blue bars indicated pathways that have been activated while red bars indicated inhibition (FDR < 0.05, *pGFWER > 0.05).

**FIGURE 8:**
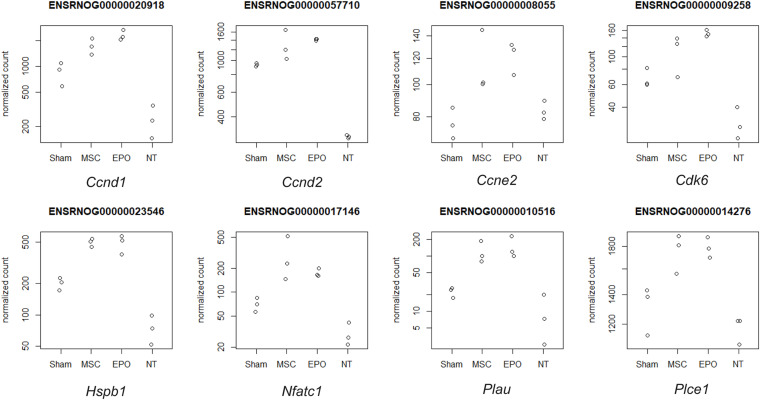
Downstream effector genes related to proliferation and growth were extracted from statistically significant pathways that were detected with SPIA. Eight genes were found to be statistically significant (*P*_adj_ < 0.05) in the MSC^EPO^-treated group. Namely, *Ccnd1*, *Ccnd2*, *Ccne2*, *Cdk6*, *Hspb1*, *Nfatc1*, *Plau*, and *Plce1*. The normalized count data for the expression of each gene was plotted across all groups.

Pathway topology analysis was performed using the Signaling Pathway Impact Analysis tool to identify the most relevant pathways based on the tested conditions in this study and to determine the status of said pathways (i.e., sensory system, cellular processes, and environmental information processing from KEGG). Phototransduction, which was one of the key pathways of this study, was found to be inhibited in the sham group (Figure 7). In both the MSC and MSC^EPO^ group, however, it was activated. Necroptosis, a form of cell death mechanism, was activated in the sham group. Although this pathway was not significant in the other groups, apoptosis was shown to be inhibited by MSC. Not only that, cellular senescence was also inhibited. This indicated that cell proliferation and growth may have taken place. Unlike the MSC group, MSC^EPO^ treatment did not reveal any significant inhibition of cell death pathways. However, the PI3K-Akt pathway was activated, and it is involved in cell survival. Other notable pathways that were statistically significant in this analysis include JAK-STAT, NF-κB, TNF, and cytokine-cytokine receptor interactions. The details were tabulated in Table 3. Based on the statistically significant pathways that were detected using SPIA, downstream effector genes involved in cellular proliferation and growth were extracted. Eight genes were found to be statistically significant (*P*_adj_ < 0.05) in the MSC^EPO^-treated group (Figure 8). The details were tabulated in Table 4.

**TABLE 3:** After performing a pathway topology analysis with SPIA, significant KEGG pathways in sensory systems and environmental information processing were tabulated. KEGG, Kyoto Encyclopedia of Genes and Genomes; FDR, false discovery rate; pGFWER, Bonferroni adjusted global p-values.

Group	Map	Pathway	KEGG	FDR	pGFWER	Status
Sham-treated	Sensory systems	Phototransduction	rno04744	3.47E-06	1.84E-05	Inhibited
	Cell growth and death	Necroptosis	rno04217	0.001	0.014	Activated
	Signaling pathway	JAK-STAT signaling pathway	rno04630	0.002	0.030	Inhibited
	Signaling pathway	PI3K-Akt signaling pathway	rno04151	0.003	0.069	Activated
	Signaling pathway	MAPK signaling pathway	rno04010	0.006	0.146	Activated
	Signal molecules	ECM-receptor interaction	rno04512	0.012	0.382	Activated
	Cell motility	Regulation of actin cytoskeleton	rno04810	0.015	0.500	Activated
	Signaling pathway	HIF-1 signaling pathway	rno04066	0.018	0.704	Activated
	Signaling pathway	NF-κB signaling pathway	rno04064	0.030	1.000	Activated
	Signaling pathway	Sphingolipid signaling pathway	rno04071	0.032	1.000	Activated
	Signaling pathway	Cgmp-PKG signaling pathway	rno04022	0.044	1.000	Activated
	Signal molecules	Neuroactive ligand-receptor interaction	rno04080	0.047	1.000	Activated
	Signaling pathway	Phospholipase D signaling pathway	rno04072	0.048	1.000	Activated
	Cell growth and death	Cellular senescence	rno04218	0.053	1.000	Activated
	Signal molecules	Cytokine-cytokine receptor interaction	rno04060	0.089	1.000	Activated
MSC-treated	Signal molecules	Cytokine-cytokine receptor interaction	rno04060	4.12E-26	4.12E-26	Activated
	Signaling transduction	NF-κB signaling pathway	rno04064	4.69E-09	1.17E-07	Activated
	Signaling transduction	JAK-STAT signaling pathway	rno04630	2.33E-06	8.63E-05	Inhibited
	Signaling transduction	TNF signaling pathway	rno04668	1.33E-05	0.001	Activated
	Cell death and growth	Cellular senescence	rno04218	5.47E-05	0.002	Inhibited
	Sensory systems	Phototransduction	rno04744	0.000	0.016	Activated
	Cell death and growth	Apoptosis	rno04210	0.008	0.468	Inhibited
	Cell death and growth	Cell cycle	rno04110	0.008	0.591	Inhibited
	Cell death and growth	Necroptosis	rno04217	0.010	0.644	Activated
	Cell motility	Regulation of actin cytoskeleton	rno04810	0.012	0.763	Activated
	Signaling transduction	Rap1 signaling pathway	rno04015	0.024	1.000	Inhibited
	Signaling transduction	HIF-1 signaling pathway	rno04066	0.028	1.000	Inhibited
	Signaling transduction	PI3K-Akt signaling pathway	rno04151	0.033	1.000	Inhibited
	Signaling transduction	MAPK signaling pathway	rno04010	0.057	1.000	Activated
MSC^EPO^-treated	Signal molecules	Cytokine-cytokine receptor interaction	rno04060	1.04E-08	3.13E-08	Activated
	Sensory systems	Phototransduction	rno04744	7.06E-07	3.53E-06	Activated
	Cell motility	Regulation of actin cytoskeleton	rno04810	0.000	0.001	Activated
	Signaling transduction	PI3K-Akt signaling pathway	rno04151	0.004	0.042	Activated
	Signaling pathway	Phospholipase D signaling pathway	rno04072	0.022	0.438	Activated
	Signaling transduction	Cgmp-PKG signaling pathway	rno04022	0.022	0.447	Inhibited
	Signal molecules	ECM-receptor interaction	rno04512	0.025	0.614	Activated

**TABLE 4:** After performing a pathway topology analysis with SPIA, the 8 downstream effector genes in proliferation and growth, that were statistically significant (*P*_adj_ < 0.05) in the MSC^EPO^-treated group, were extracted and tabulated.

ID	Symbol	Description	Log_2_FC	*P*_adj_
ENSRNOG00000020918	*Ccnd1*	Regulation of cyclin-dependent protein serine/threonine kinase activity	1.2810	4.13E-06
ENSRNOG00000057710	*Ccnd2*	Regulation of cyclin-dependent protein serine/threonine kinase activity	0.5203	0.0002
ENSRNOG00000008055	*Ccne2*	Regulation of cyclin-dependent protein serine/threonine kinase activity	0.5108	0.0036
ENSRNOG00000009258	*Cdk6*	Regulation of transcription by RNA polymerase II	0.9829	0.0041
ENSRNOG00000023546	*Hspb1*	Proteasome complex	1.2080	1.19E-06
ENSRNOG00000017146	*Nfatc1*	G1/S transition of mitotic cell cycle	0.9934	0.0018
ENSRNOG00000010516	*Plau*	Angiogenesis	2.5007	8.13E-07
ENSRNOG00000014276	*Plce1*	Golgi membrane	0.3904	0.0004

## Discussion

The current study aimed to determine the impact of transplanted erythropoietin-expressing MSCs on the fate of sodium iodate-induced retinas on a functional and transcriptional level. During this study period, we established that intravitreally transplanted human MSCs^EPO^ were able to graft onto the retina. The tracking of transplanted cells was done both by observation of green fluorescence as a result of the expression of GFP in the lentiviral construct, and staining for human stem cells cytoplasm by using anti-STEM121 antibodies in the rat retinal tissue (Figures 1, 2). These antibodies are extensively used in detecting the migration and engraftment of human stem cell transplants in animal models (Chao et al., 2017; Singh et al., 2019). The results demonstrated a very dim expression of GFP, probably due to GFP-induced cytotoxicity in MSCs (Ansari et al., 2016) or failure of the construct itself to express the tag in the *in vivo* system (Chao et al., 2017). The cells were able to persist in the host’s microenvironment for an extended period until the end of the experiment on day 30. Although this engraftment was not investigated past day 30, other studies, like that of Tzameret et al. (2015), were able to show a similar resilience of human MSCs in the rodent vitreous space more than 4 weeks post-transplant. As shown in Figures 1, 2, transplanted MSCs^EPO^ formed a sheet of cells that adhered to the retina, which resulted in the thickening of the tissue. This was commonly observed in other rodent studies that have employed the intravitreal route of MSC transplantation (Labrador Velandia et al., 2018; Hu et al., 2020). Those studies showed that MSCs mostly remain in the vitreous space and may form membranes rather than penetrate the inner retinal layers. This led to a reduction in retinal cell death from the release of paracrine factors, but such layered structures have also been shown to impede visual function and even cause retinal detachment in humans (Satarian et al., 2017). Interestingly, this was found to be donor-specific. To determine if the stem cells in our study had such adverse effects on retinal function, further experiment using electroretinography was carried out.

Electroretinography is a test that detects electrophysiological responses in the eye under a light stimulus (Marmor et al., 2009). In the current study, 5 flash intensities between 0.003 and 3.0 cd.s/m^2^ were used to evoke the ERG responses. This range of illumination is comparable to that of moonlight and a dimly-light office (Fred Schubert, 2018). Both MSC and MSC^EPO^-treated groups were able to produce detectable ERG b waves, which were indicative of successful phototransduction (Figure 3; Qu et al., 2017). The MSC-treatment showed efficacy in all the tested ERG parameters, however, the improvement in visual functions was diminishing by week 4. Conversely, MSC^EPO^-treatment effect was able to maintain the improvement in visual functions throughout the study even though it was only significant using the brightest flash intensity. Still, the preservation of visual functions by MSCs^EPO^ group meant that it could perform better with long term benefits. Extending the study by a few months may show a prolonged preservation of visual functions in the MSCs^EPO^ group while the MSC group will likely experience a further deterioration of the retina. This was not explored in the present study. However, a study done by Guan et al. (2013) showed that transplanted rat MSCs^EPO^ produced spectacular results 8 weeks after subretinal injection. From these findings, it is possible that EPO might promote MSC survival in a harsh microenvironment, thereby prolonging their therapeutic effect in the retina as demonstrated by our ERG data. To comprehend the mechanisms on how both MSC and MSCs^EPO^ could effect an improvement in visual function in rats, a transcriptomics analysis on the rat tissue itself was performed after the transplantation.

Following RNA-seq, an exploratory analysis was conducted to visualize the clustering of the individual samples (Figure 4). This was to ensure that large sample dispersions do not affect differential gene expression. By observing the pattern of gene expressions affected by MSCs and MSCs^EPO^, there was an obvious shift toward strongly expressing differentially expressed genes (DEGs) (Figure 5). An over-representation analysis (ORA) was then performed to profile these DEGs into a more meaningful representation of biological data and then identify the most over-represented processes. Firstly, DEGs in sham were compared with those from NT to identify biological processes that were highly affected by the administration of NaIO_3_. The analysis yielded a set of GO-enriched biological processes that were mostly involved in the immune response (Table 1). Cell activation (GO:0001775) was the top affected process, and this is usually due to exposure to an active ligand that binds to its respective receptor. This process includes cellular differentiation, growth, and motility. Not only that, but the sham-treated group also revealed several immune processes. This is likely due to the cytotoxic effects of sodium iodate (NaIO_3_) on the retina. NaIO_3_ is an oxidizing agent that specifically affects the RPE (Tao et al., 2013). This form of stress results in a deregulation of its physiological processes, and thus causing RPE cell death, subsequent photoreceptor cell death, and neurotransmission impairment (Donato et al., 2020, 2021; Scimone et al., 2020). Since the RPE and photoreceptors act as a blood-retinal barrier, a breach will cause the eye to lose its immune-privilege. As such, there will be an influx of migrating immune cells into the eye that will be activated by the presence of dead retinal cells (Gordon and Plüddemann, 2018). This pattern of gene expression was similar for the MSC and MSC^EPO^ groups, however, the immune response in MSC-treated samples was several magnitudes higher than MSC^EPO^. This may have explained why the efficacy of MSCs began to drop after week 2 of the study, as shown in the ERG test results. Although MSCs are known to modulate the immune response, a high cell dose can trigger immunity in a xenogeneic environment (Hwang et al., 2020), as displayed in this study. We recommend that future studies increase the immunosuppressant dosage above what was used in this study. Interestingly, ORA revealed that MSCs^EPO^ was not as immunoreactive. This was likely because of EPO, as its administration before kidney transplants was well associated with increased graft survival due to a possible link between EPO-receptor signaling and diminished T-cell responses (Cravedi et al., 2014).

A majority of the processes detected with ORA involved the immune response, which made it challenging to identify other notable pathways despite our functional tests revealing improved ERG b wave amplitudes. As such, a GSEA analysis was performed to determine whether healing and regeneration played a role in this study. The results yielded an enrichment of wound healing processes that were negatively regulated in the sham group (Figure 6). Conversely, positive regulation of wound healing was enriched in both the MSCs and MSC^EPO^ groups. Not only that, animal organ regeneration was also enriched. The nominal enrichment scores were tabulated in Table 2. This pattern of enrichment suggested that the transplantation of MSCs and MSCs^EPO^ could promote retinal regeneration and this, in turn, will lead to improved visual functions. To show how these cells impacted key pathways involved in cellular proliferation, growth, and phototransduction, an SPIA analysis was performed. By observing the results in Figure 7, the phototransduction pathway was inhibited in the sham group but activated in the MSC and MSC^EPO^ groups. The sham group also exhibited an activation of the necroptosis pathway, which is the main cell death pathway that is triggered by NaIO_3_ in the retina (Hanus et al., 2016; Zhao et al., 2017). This finding complemented the work of other studies in necroptotic retinal cell death. In the MSC group, other than phototransduction, several other pathways were activated. This included cytokine-cytokine reactions, which were mainly activated by immune-related genes (Supplementary Table 1). NF-κB (nuclear factor kappa B) and TNF (tumor necrosis factor) signaling were also activated. Both pathways play a crucial role in the immune response (Zhang et al., 2017; Varfolomeev and Vucic, 2018). Although TNF signaling is involved with cellular proliferation, the co-significance of the previously mentioned pathways in the MSC group, coupled with ORA, highly suggested the involvement of active immune cells that possibly reduced the efficacy of MSC treatment over time. Still, the treatment was effective enough to inhibit the cellular senescence and apoptosis pathways. However, the JAK-STAT (Janus kinase-signal transducer and activator of transcription) pathway was inhibited in both the sham and MSC groups. As it plays a role in retinal cell survival, the inhibition of this pathway may have reflected the degenerative state of these tissues at the time of this study (Huang et al., 2007; Beach et al., 2017; Lozano et al., 2019).

In the MSC^EPO^ group, a lack of the core pathways that mediated immune responses in the MSC group was not detected here. This suggested a possible reduction in said response by MSCs^EPO^ via enhanced immunomodulation. The actin cytoskeleton pathway was also activated, and it is primarily involved in cell development, motility, and adhesion. Migration and homing are defining features of MSC-mediated regeneration, and the activation of this pathway could be a reflection of what transpired in the retina of the host after transplantation (Ding et al., 2017; Tang and Gerlach, 2017). Most notably, however, was the activation of the PI3k-Akt (phosphatidylinositol 3-kinase/protein kinase B) pathway in the MSC^EPO^ group. This pathway primarily governs cellular development in erythroid progenitors through EPO and EPO receptor binding (Sivertsen et al., 2006; Kuhrt and Wojchowski, 2015). This receptor is also expressed in the central nervous system (Ma et al., 2016) and the retina (García-Ramírez et al., 2008; Shah et al., 2009; Ding et al., 2016). The activation of the PI3k-Akt pathway by EPO was found to confer neuroprotective and anti-apoptotic effects in neurons (Si et al., 2019). This was due to the modulation in gene expressions involving Bcl-xL, Bax, and BAD (Shen et al., 2010). The application of EPO in treating retinal degeneration is not a new concept, as findings have indeed shown its efficacy in protecting the retina (King et al., 2007; Shen et al., 2014; Luo et al., 2015). Several studies have also been done on the pretreatment of MSCs with EPO, and the results have led to an enhancement in regenerative capabilities (Lu et al., 2016; Zhou et al., 2018; Imam and Rizk, 2019). However, genetically modifying MSCs to express EPO for increased therapeutic synergy is not widely explored. Guan et al. (2013) were able to show the potential of rat MSCs^EPO^ in rescuing NaIO_3_-induced retinal degeneration. A separate study done by Lin et al. (2019) showed that in a liver fibrosis model, mouse MSCs^EPO^ conferred enhanced anti-fibrotic efficacy. By analyzing the transcriptomic profile after MSC^EPO^-treatment, we were able to extract several pro-survival genes that were significantly expressed (Figure 8). A majority of these genes, such as *Ccnd1*, *Ccne2*, and C*dk6* play a role in cell cycle progression and growth (Table 4). It has been shown that by downregulating CCND1 and CDK6 expression in human cell lines, cell cycle arrest was successfully induced (Maacha et al., 2020). Exploring these genes as potential candidates for MSC modification could result in enhanced MSC graft survival. *Plau*, which has chief functions in angiogenesis important for tissue regeneration, was also highly expressed (Tao et al., 2016; Maacha et al., 2020).

Based on the findings of the present study, the transcriptome analysis and *in vivo* tests showed that paracrine effects played a major role in MSC^EPO^-mediated regeneration through the upregulation of pro-survival pathways such as PI3K-Akt signaling. In one study, we treated the Y79 retinal cell line with a conditioned medium obtained from MSCs^EPO^ before glutamic acid exposure. The results indicated that the presence of EPO could further enhance protection to the oxidative stress incurred onto the Y79 retinal cells compared with the conditioned medium obtained from MSC culture alone (Ding et al., 2018). In another experiment, we showed that a conditioned medium obtained from MSCs^EPO^ could immediately prevent retinal pigmented epithelial (ARPE) cell line death upon exposure to sodium iodate (Koh et al., 2021). Our *in vitro* results supported the notion that a combination of EPO and MSC paracrine factors could further enhance the regenerative properties of MSCs in reversing retinal damage in the current study, as indicated by the improvement in the ERG data. In addition, we also performed immunohistochemical staining to determine if these stem cells have directly differentiated into other retinal neurons or the RPE. However, there was no direct cell differentiation detected from the stem cells. This was in contrast to our previous study, where we showed that the stem cells differentiated into photoreceptors, bipolar cells, and Muller glia in the rat retina (Leow et al., 2015). We performed staining with the markers, PKCα, rhodopsin, and RPE65, in the present study but were unable to detect MSCs^EPO^ differentiation *in vivo*. This was probably due to the shorter treatment duration of MSCs^EPO^ before the rats were sacrificed. Prolonging the treatment duration similar to our previous study might lead to the detection of differentiated retinal cell markers. Of note, allowing stem cells to replicate in a foreign microenvironment in long term may also possibly lead to teratoma formation, which will inevitably cause further loss of retina function. This aspect was not investigated in our study since we observed improvement in retinal functions.

Our current data showed that there is a possible enhancement of immunomodulatory capabilities in MSCs^EPO^. Further downstream analyses such as immune cell-staining will shed further light regarding the mounted immunity against the xenotransplants. This can be further validated using qPCR and western blot. By increasing the depth of coverage to enhance NGS sensitivity, transplanted human MSC^EPO^ gene expression can be detected and analyzed to complement the retinal transcriptomic profile. The amount of EPO expressed by MSCs^EPO^ in the retina may not have been sufficient to better improve retinal survival, and the lack of this quantitative data in the present study is another limitation that should be addressed with further analyses. The EPO level could perhaps be measured in extracted vitreous fluid or blood. In a nutshell, our study has shown that MSCs^EPO^ hold tremendous potential in protecting the retina from retinal degeneration. Furthermore, the expression data was complemented by our functional ERG data. This meant that the presence of ERG b wave amplitudes *in vivo* validated the activation of the phototransduction pathway *in silico*. All in all, it is hoped that these findings could aid researchers in identifying candidate genes for MSC modification or producing novel MSC enhancement methods in the field of regenerative medicine.

## Conclusion

In summary, the findings of our study showed the tremendous potential of MSCs^EPO^ in protecting the retina from retinal degeneration. The cells were able to persist in the host for at least 30 days during the study. Follow-up ERG tests post-transplant showed an improvement in ERG b wave amplitudes. A series of transcriptomics analyses then revealed enrichment of pathways involving wound healing and regeneration in the MSC^EPO^-treated group. This likely resulted in the improvement of visual function, as shown by the activation of key pathways such as phototransduction. The ERG data served as a means to validate this *in silico* result. The large number of immune-related pathways detected in the MSC-treated group was not present in the MSC^EPO^-treated group. This suggested that erythropoietin may modulate the immune reaction in the rat model. Furthermore, we were able to extract significantly expressed pro-survival genes as possible candidates for future study.

## Data Availability Statement

The datasets presented in this study can be found in the GEO public repository. The names of the repository/repositories and accession number(s) can be found below: https://www.ncbi.nlm.nih.gov/, GSE164152.

## Ethics Statement

The animal study was reviewed and approved by Universiti Kebangsaan Malaysia Animal Ethics Committee (UKMMAEC).

## Author Contributions

MPL and SKS conceived the experimental study design. AEHK and HAA carried out the research study and composed the manuscript. MR, CL, and MH aided in the electroretinography tests and analysis. TKY, Bastion MLC, NMH, HMI, AF, and MKA supported the study design, and analyzed and commented on the data. MPL and SKS edited the manuscript. All authors were involved in reviewing the manuscript.

## Conflict of Interest

The authors declare that the research was conducted in the absence of any commercial or financial relationships that could be construed as a potential conflict of interest.

## Supplementary Material

Supplementary Table 1 After performing a pathway topology analysis with SPIA, significant KEGG pathways in sensory systems, cellular processes, and environmental information processing were tabulated. KEGG, Kyoto Encyclopedia of Genes and Genomes; pGFDR, false discovery rate; pGFWER, Bonferroni adjusted global *p*-values; NDE, observed number of genes; tA, observed total accumulation; pPERT, perturbations accumulation *P* value; pG, *P* value. KEGGLINK provides a web link to KEGG displaying the current dataset in relation to highlighted pathways.
