# Corrigendum: Transplanted Erythropoietin-Expressing Mesenchymal Stem Cells Promote Pro-survival Gene Expression and Protect Photoreceptors From Sodium Iodate-Induced Cytotoxicity in a Retinal Degeneration Model

**DOI:** 10.3389/fcell.2021.764504

**Published:** 2021-09-16

**Authors:** Avin Ee-Hwan Koh, Hiba Amer Alsaeedi, Munirah Binti Abd Rashid, Chenshen Lam, Mohd Hairul Nizam Harun, Min Hwei Ng, Hazlita Mohd Isa, Kong Yong Then, Mae-Lynn Catherine Bastion, Aisha Farhana, Mohammad Khursheed Alam, Suresh Kumar Subbiah, Pooi Ling Mok

**Affiliations:** ^1^Department of Clinical Laboratory Sciences, College of Applied Medical Sciences, Jouf University, Sakaka, Saudi Arabia; ^2^Department of Biomedical Sciences, Faculty of Medicine and Health Sciences, Universiti Putra Malaysia, Selangor, Malaysia; ^3^Department of Ophthalmology, Faculty of Medicine, Universiti Kebangsaan Malaysia Medical Centre, Kuala Lumpur, Malaysia; ^4^Tissue Engineering Centre, Universiti Kebangsaan Malaysia Medical Center, Kuala Lumpur, Malaysia; ^5^Department of Orthodontics, College of Dentistry, Jouf University, Sakaka, Saudi Arabia; ^6^Department of Medical Microbiology, Universiti Putra Malaysia, Serdang, Malaysia; ^7^Genetics and Regenerative Medicine Research Group, Universiti Putra Malaysia, Serdang, Malaysia; ^8^Centre for Materials Engineering and Regenerative Medicine, Bharath Institute of Higher Education and Research, Chennai, India

**Keywords:** mesenchymal stem cells, erythropoietin, sodium iodate, transcriptome, photoreceptors, pro-survival genes

In the published article, there was an error in affiliation 6 and 8. Instead of “Department of Medical Microbiology and Parasitology, Universiti Putra Malaysia, Serdang, Malaysia.” it should be “Department of Medical Microbiology, Universiti Putra Malaysia, Serdang, Malaysia.” And “Department of Biotechnology, Bharath Institute of Higher Education and Research, Chennai India.” should be “Centre for Materials Engineering and Regenerative Medicine, Bharath Institute of Higher Education and Research, Chennai, India.”

In the published article, there was an error regarding the affiliation for Avin Ee-Hwan Koh. The correct affiliations should be “Department of Clinical Laboratory Sciences, College of Applied Medical Sciences, Jouf University, Sakaka, Saudi Arabia.” And “Department of Biomedical Sciences, Faculty of Medicine and Health Sciences, Universiti Putra Malaysia, Selangor, Malaysia.”

The authors apologize for these errors and state that this does not change the scientific conclusions of the article in any way. The original article has been updated.

## Publisher's Note

All claims expressed in this article are solely those of the authors and do not necessarily represent those of their affiliated organizations, or those of the publisher, the editors and the reviewers. Any product that may be evaluated in this article, or claim that may be made by its manufacturer, is not guaranteed or endorsed by the publisher.

